# Transformation of the drug ibuprofen by *Priestia megaterium*: reversible glycosylation and generation of hydroxylated metabolites

**DOI:** 10.1007/s11356-025-36393-5

**Published:** 2025-04-21

**Authors:** Tjorven Hinzke, Rabea Schlüter, Annett Mikolasch, Daniela Zühlke, Patrick Müller, Robert Kleditz, Katharina Riedel, Michael Lalk, Dörte Becher, Halah Sheikhany, Frieder Schauer

**Affiliations:** 1 Institute of Microbiology, University of Greifswald, Felix-Hausdorff-Straße 8, 17489 Greifswald, Germany; 2 Helmholtz Institute for One Health (HIOH), Helmholtz-Centre for Infection Research (HZI), 17489 Greifswald, Germany; 3 Institute of Biochemistry, University of Greifswald, Felix-Hausdorff-Straße 4, 17489 Greifswald, Germany

**Keywords:** Biodegradation, Micropollutants, Non-steroidal anti-inflammatory drugs (NSAIDs), *Bacillus subtilis*, Metabolism, Xenobiotics

## Abstract

As one of the most-consumed drugs worldwide, ibuprofen (IBU) reaches the environment in considerable amounts as environmental pollutant, necessitating studies of its biotransformation as potential removal mechanism. Here, we screened bacteria with known capabilities to degrade aromatic environmental pollutants, belonging to the genera *Bacillus*, *Priestia* (formerly also *Bacillus*), *Paenibacillus*, *Mycobacterium*, and *Cupriavidus*, for their ability to transform ibuprofen. We identified seven transformation products, namely 2-hydroxyibuprofen, carboxyibuprofen, ibuprofen pyranoside, 2-hydroxyibuprofen pyranoside, 4-carboxy-α-methylbenzene-acetic acid, 1-[4-(2-hydroxy-2-methylpropyl)phenyl]ethanone, and 2-hydroxyibuprofenmethyl ester. Based on our screening results, we focused on ibuprofen biotransformation by *Priestia megaterium* SBUG 518, to identify structures of transformation products, and to shed light on the drug’s impact on bacterial physiology. Biotransformation reactions by *P. megaterium* SBUG 518 involved (A) the hydroxylation of the isobutyl side chain at two positions, and (B) conjugate formation via esterification with a sugar molecule of the carboxylic group of ibuprofen and an ibuprofen hydroxylation product. Glycosylation seems to be a detoxification process, since the ibuprofen conjugate (ibuprofen pyranoside) was considerably less toxic than the parent compound to *P. megaterium* SBUG 518. Based on proteome profile changes and inhibition assays, cytochrome P450 systems appear to be central for ibuprofen transformation in *P. megaterium* SBUG 518. The toxic effect of ibuprofen appears to be caused by interference of the drug with different physiological pathways, especially sporulation.

**Supplementary Information:**

The online version contains supplementary material available at 10.1007/s11356-025-36393-5.

## Importance

Ibuprofen is a highly consumed drug, and reaches the environment in considerable quantities as an environmental pollutant. Therefore, it is of great interest how microorganisms transform this drug, and how microbial physiology is impacted by it. Here, we screened several bacteria for their ability to transform ibuprofen. *Priestia megaterium* SBUG 518 emerged as highly capable for ibuprofen transformation and was therefore studied in greater detail. We show that *P. megaterium* SBUG 518 transforms ibuprofen via two main pathways, hydrolyzation and reversible conjugation. These pathways resemble those in humans. Ibuprofen likely impacts the physiology of *P. megaterium* SBUG 518 on several levels, including spore formation. Taken together, *P. megaterium* SBUG 518 is well suited as a model organism to study bacterial ibuprofen metabolism.

## Introduction

Pharmaceuticals, albeit intended as being beneficial to diagnose and treat illnesses, can not only have harmful side effects for humans, but can also be detrimental for microorganisms, animals, and ecosystems (e.g., Cleuvers 2003; Daughton & Ternes 1999; Heckmann et al. 2007; Markandya et al. 2008; Oaks et al. 2004; Saravanan et al. 2012).

Ibuprofen (IBU), a pain-mitigating and non-steroidal anti-inflammatory drug (NSAID), is the most commonly used analgesic in Germany (Sarganas et al. 2015) and the world’s third-most consumed drug (Chopra and Kumar 2020). A main source of environmental contamination are wastewater treatment plant effluents: despite that IBU is removed from wastewater with comparatively high efficiency of around 90 % or more (e.g., Gros et al. 2010; Ternes 1998; Yu et al. 2006), it is frequently detected in the effluents, as well as in surface waters (Weigel et al. 2004; Wojcieszyńska et al. 2022).

While IBU transformation and degradation in the environment are thus of great interest, microbial IBU transformation and degradation pathways are still largely unknown (see Mulkiewicz et al. (2021) for a comprehensive review). Studies regarding IBU transformation by microorganisms include foremost whole-community degradation studies in bioreactors (Maeng et al. 2013; Calero-Díaz et al. 2017), and activated sludge communities (Urase and Kikuta 2005; Peng et al. 2019). Some bacterial strains which degrade IBU are described, including *Patulibacter* sp. I11 (Almeida et al. 2013), *Nocardia* sp. (Chen and Rosazza 1994), *Sphingomonas* sp. Ibu-2 (Murdoch and Hay 2005), *Variovorax* sp. Ibu-1 (Murdoch and Hay 2015), *Rhizorhabdus* (*Sphingomonas*) *wittichii* MPO218 (Aulestia et al. 2021, 2022), *Sphigopyxis granuli* (Aguilar-Romero et al. 2021), *Rhodococcus cerastii* (Ivshina et al. 2021), *Bacillus thuringiensis* B1 (Marchlewicz et al. 2016), and *Rhizobium daejeonense* IBU_18 (Pápai et al. 2023). Additionally, while IBU has antimicrobial effects (Elvers and Wright 1995; Obad et al. 2015; Zimmermann and Curtis 2017), the impact of this drug on bacterial physiology and metabolism still needs to be elucidated.

To shed light on microbial IBU degradation pathways and physiological reactions to this drug in bacteria, we conducted a screening experiment to test several bacterial strains for their ability to transform IBU. A total of seven bacterial strains were chosen for testing based on known capability for environmental pollutant degradation. These strains belonged to the genera *Bacillus*, *Priestia*, *Paenibacillus*, *Mycobacterium*, and *Cupriavidus*. Incubations with *Priestia megaterium* SBUG 518 (formerly *Bacillus megaterium* SBUG 518; Gupta et al. 2020) yielded the most transformation products, and we performed further in-depth analyses with this strain. Our analyses encompassed elucidating IBU transformation pathways, as well as analysing the strains’ global proteomic responses to IBU.

We here show that *P. megaterium* SBUG 518 hydroxylates IBU, and, in addition, glycosylates not only IBU, but also at least one IBU oxidation product. We demonstrate that the direct glycosylation of IBU is reversible.

Furthermore, changes in the proteomic profile of *P. megaterium* SBUG 518 suggest that a *P. megaterium*-specific cytochrome P450 system is involved in IBU transformation, and that IBU interferes with sporulation, amino acid and fatty acid metabolism, and potentially with the oxidative stress response, providing a molecular basis for the toxic effect of IBU on *P. megaterium* SBUG 518.

## Material and methods

### Strains

For screening experiments, we tested seven bacterial strains with known transformation capabilities for their effectiveness to transform IBU (Table 1). The strains are deposited into the strain collection of the Department of Biology of the University of Greifswald (SBUG).

**Table 1:** Bacterial strains used in this study, their origin and transformation capabilities of these or related strains. ATCC: American Type Culture Collection; DSM: Deutsche Sammlung von Mikroorganismen

**Str**a**in**	Origin/original strain number	Transformation capabilities
*Priestia megaterium* SBUG 518	ATCC 2007	Oxidation of phenanthrene, fluoranthrene, pyrene (Carmichael and Wong 2001) Oxidation and hydroxylation of betulinic acid (Chatterjee et al. 2000) Dichloroanalines as C- and energy source (Yao et al. 2011)
*Priestia megaterium* SBUG 1979	R. Biedendieck, Technical University Braunschweig, Institute of Microbiology, 01/2014	
*Bacillus pumilus* SBUG 1800	isolated from Rassower Strom (Brack 2010)	Vanillin degradation, including ring cleavage (Hua et al. 2007) Ferulic acid decarboxylation (Lee et al. 1998)
*Bacillus thuringiensis* SBUG 1431	DSM 350	Dimethyl phthalate as C source (Brar et al. 2009)
*Cupriavidus basilensis* SBUG 290	(Becher 1997)	Dibenzofuran degradation (Becher et al. 2000) Bisphenol transformation and ring cleavage (Henning 2011; Zühlke 2013)
*Mycobacterium neoaurum* SBUG 109	(Mikolasch et al. 2003)	Transformation of ibuprofen and acetaminophen (Garbe 2008; Dräger 2012) Growth on pristane (Nhi-Cong et al. 2009)
*Paenibacillus apiarius* SBUG 1947	Isolated from Lake Balschasch (Meene et al. 2022)	Growth on dibenzofuran (Iida et al. 2006)

### Bacterial culturing and incubation for biotransformation experiments and toxicity assays

*Culturing.* Culture conditions were chosen according to the respective organisms’ physiology and expert knowledge (data not shown). We cultivated all strains of the genera *Bacillus*, *Priestia*, and *Paenibacillus* in 500-mL flasks, containing 100 mL of complex medium nutrient broth Nr. 2 (NB II) for 24 h at 30 °C and 180 rpm on a rotary shaker (HT FORS, Infors AG, Bottmingen, Switzerland). Additionally, both strains of *P. megaterium* SBUG 518 were cultivated in complex medium Lysogeny broth (LB), and also used in the logarithmic phase of growth after 5 h of incubation in NB II with an optical density OD_500nm_ of 1.0 to 1.5. Cultivation of *C. basilensis* with biphenyl was carried out following Zühlke et al. (2017), with the minor modification that the main culture was inoculated with only 5 mL cell suspension in 100 mL NB II. *M. neoaurum* was cultivated on agar plates with tetradecane for 4 d at 30 °C, using material of five plates for biotransformation experiments as described by Nhi-Cong et al. 2009. Cells were transferred into 75 mL mineral salts medium for bacteria (MMb according to Hundt et al. (1998); pH 6.3; MMb does not contain a C source bacterial growth), supplemented with IBU (see below).

*Transformation assays.* Transformation experiments with IBU were performed in 500 mL flasks containing 100 mL MMb and 0.005% (0.05 mg mL^−1^; equivalent to 219 µm) IBU. For incubation experiments in the presence of glucose, 0.1% glucose was added from a 50% (w/v) aqueous glucose stock solution. After cultivation, we harvested the cells by centrifugation (11,270 × g, 10 min, 4 °C), washed them twice with MMb and resuspended them in a small amount of MMb. We then added this cell suspension to the flasks with MMb and IBU to reach an optical density at 500 nm (OD_500nm_) of 3.0, corresponding in the case of *P. megaterium* SBUG 518 to a cell titre of approximately 3.95 * 10^8^ (± 0.55 * 10^8^) cells mL^−1^. Incubation experiments were carried out on a rotary shaker at 30 °C and 130 rpm (VKS-75 Control, Edmund Bühler GmbH, Bodelshausen, Germany). Two types of controls were used: (i) flasks with cells in MMb without IBU and (ii) flasks with IBU in MMb without cells.

*Cytochrome P450 inhibition studies.* To study the effect of cytochrome P450 inhibition on IBU biotransformation by *P. megaterium* SBUG 518, we added 219 μM 1-aminobenzotriazole to transformation assays with 219 μM IBU (0.005%) in the presence and absence of 0.1% glucose, and performed transformation experiments as above.

*IBU toxicity assays.* To assess toxic effects of IBU and IBU pyranoside (IBU-PYR, product P3) on the growth of *P. megaterium* SBUG 518, we performed growth assays with and without these substances in NB II (pH 6.0; adjusted with HCl from an initial pH of 7.2). We decreased the pH to allow for greater IBU toxicity effects against *P. megaterium* SBUG 518. Cells were cultivated for 24 h as described above. Cells were transferred into 100 mL Erlenmeyer-flasks containing 20 mL NB II, pH 6.0 (OD_500nm_ = 0.2). Some 43.8 µM IBU (corresponding to a growth inhibitory concentration of 0.05% IBU) or 43.8 µM of product P3, respectively, were added. Controls without IBU or IBU transformation product were included in the assays. Subsequently, we cultivated the assays for 72 h at 30 °C and 180 rpm, and periodically determined the OD_500nm_.

### Analysis of IBU biotransformation with high-performance liquid chromatography (HPLC)

To study IBU biotransformation, including the formation of transformation products, over the time course of the incubations, we used 1-ml samples of culture supernatant taken under sterile conditions at selected time points. We removed cells via centrifugation (3,600 × g, 10 min, Hettich Universal 30F, Tuttlingen) and analyzed 60 µL of the resulting supernatant by HPLC using an Agilent-Technologies 1200 Series system (Santa Clara, USA). Products were separated on a LiChroCART 125-4 RP-18 end-capped 5 µm column (Merck, Darmstadt, Germany) with a solvent system of methanol and phosphoric acid (0.1%, v/v) using a linear gradient from 30% to 100% methanol over a period of 14 min at a flow rate of 1 mL min^−1^. UV-Vis spectra were recorded using a diode array detector.

### Extraction of biotransformation products

For purification of transformation products, we harvested culture supernatants from a total of 2 L of cultures by centrifugation (3,600 × g, 5 min, Hettich Universal 30F) and extracted the supernatants four times with twice the volume of ethyl acetate at pH 7 and again four times with twice the volume of ethyl acetate at pH 2. A 25% (w/v) aqueous sodium hydroxide solution was used to adjust the pH value to pH 7; the aqueous supernatant was then adjusted to pH 2 with 32% (v/v) hydrochloric acid. Organic phases were dried with anhydrous sodium sulfate. After rotary evaporation, we resolved the residues in 150 µL methanol and stored them at −20°C until further analysis.

### Chemical analysis, isolation, and identification of IBU transformation products

IBU transformation products were purified on an Agilent Technologies 1260 Infinity semi-preparative HPLC (Santa Clara, USA) with an Eclipse XDB-C18, PN 977 250-102, 21.2 × 250 mm; 7 mm column (Agilent, Santa Clara, USA), using an 8 min linear gradient of 40 to 100% methanol in acetic acid (0.1% v/v) at a flow rate of 10 mL min^−1^. UV-Vis spectra were recorded with a diode array detector. The isolated products were concentrated by rotary evaporation, the residues dissolved in methanol and stored at −20 °C until further analysis. For determining product masses, residues were transferred into weighed vials, methanol removed with a stream of gaseous nitrogen, vials weighed again and the weight difference determined.

An Agilent Technologies 1200 Series 6120 Quadrupole model was used for liquid chromatography-mass spectrometry (LC-MS) analysis. A ZORBAX SB-C18 column (2.1 x 50 mm, pore size 1.8 µm) was used for HPLC separation at a flow rate of 0.1 mL min^−1^ with a 7 min linear gradient from 10 to 100% acetonitrile in 0.1% aqueous ammonium formate. The MS was used with an electrospray ionization (API-ES) source (dry and nebulizer gas: nitrogen; drying gas flow 10.0 L min^−1^; nebulizer pressure 45 psig; drying gas temperature 350 °C; capillary voltage 4000 V). Positive and negative mass spectrometer modes were used, with the following parameters: scan range 40–600 *m/z*, fragmentor voltage 75 V, gain 1.

We used the methods described by Mikolasch et al. (2016) for the analyses of extracts by gas chromatography coupled to mass spectrometry (GC-MS). In brief, IBU biotransformation products were detected by injecting 1 µL of the extracts of the biotransformation experiments into an Agilent gas chromatograph 7890A GC System (Waldbronn, Germany) equipped with a capillary column (Agilent 1901 S-433, 30 m x 250 µm × 0.25 µm, HP-5ms column) and a mass selective detector 5975C inert XL EI/CI MSD with a quadrupole mass spectrometer. For details, please refer to the Supplementary Methods.

The nuclear magnetic resonance (NMR) spectra of four products (P1, P3, P5, P6, and P7; see Results and Supplementary Information) dissolved in dimethyl sulfoxide (DMSO)-d6 were obtained on a Bruker Avance-II instrument (Bruker Biospin GmbH, Rheinstetten, Germany) at 600 MHz (^1^H, ^13^C, heteronuclear multiple bond correlation (HMBC), heteronuclear single quantum coherence (HSQC)).

### Chemicals

IBU sodium salt (molecular weight of 228.26 g mol^−1^), 2-hydroxyibuprofen (2-OH-IBU) and carboxyibuprofen (CBX-IBU) were obtained from Sigma-Aldrich (Steinheim, Germany). All chemicals and solvents used were of the highest purity available.

### Analysis of impacts of IBU on the proteome of *P. megaterium* SBUG 518

*Proteomics sample preparation and measurement.* To analyse changes in the *P. megaterium* SBUG 518 proteome profile after incubation with IBU, we took 10 mL samples of *P. megaterium* SBUG 518 IBU transformation assays conducted with stationary-phase cells and of control incubation without IBU after 1 h and 24 h of incubation. We immediately placed samples on ice, harvested cells by centrifugation (10,015 × g, 5 min, 4 °C, Heraeus Biofuge Primo R, Thermo Scientific), washed cell pellets twice in TE buffer (10 mM Tris, 2 mM EDTA, pH 7.5) and subsequently resuspended the cells in TE buffer with 1% (w/v) Triton-X-100. Proteins were extracted by bead-beating and acetone precipitation. Subsequently, proteins were digested into peptides using 1D-SDS-PAGE (for details, please see Supplementary Methods).

Liquid chromatography-tandem mass spectrometry (LC-MS-MS) analysis was performed using a nanoACQUITY^TM^-UPLC^TM^-System (Waters, Milford, USA) combined with a linear trap quadrupole (LTQ)-Orbitrap mass spectrometer (Thermo Fisher Scientific, Waltham, USA). For details, please refer to the Supplementary Methods.

*Protein identification.* To identify detected proteins, raw spectra were searched against a forward-reverse database of *P. megaterium* ATCC 14581 (20.11.2017, Uniprot; annotations updated 16.03.2024) with added common laboratory contaminants. Sorcerer^TM^-SEQUEST^®^ and Scaffold_4 were used, with the following parameters: trypsin (KR), maximum two omitted cleavage sites, precursor mass monoisotopic, precursor mass range 400–4,500 Da, 1 Da fragment mass accuracy, b- and y-ion series, peptide mass tolerance 10 ppm, variable oxidation of methionine (15.99 Da) with a maximum of four modifications per peptide. The search result was filtered with “XCorr versus charge state”-filters as follows: 2.2 for twofold charged, 3.3 for threefold and 3.75 for fourfold and higher charged ions and DeltaCn 0.1.

*Statistical evaluation and functional annotation of proteomics results.* Proteins were quantified by calculating Normalized Spectral Abundance Factors (NSAFs; Zybailov et al. 2006). Statistical analysis was performed in R (R Core Team, 2023) using the package limma v. 3.50.3 (Ritchie et al. 2015). Correction of *p*-values for multiple comparisons was done using Benjamini-Hochberg correction (*α*= 0.05; Benjamini and Hochberg 1995) implemented in limma. Additionally, we required proteins to have at least a 2-fold abundance change (i.e., a log_2_ fold change of <-1 or > 1) in the IBU incubation vs. control at the same sampling timepoint to be considered biologically significantly changed. For visualization, we employed the package EnhancedVolcano 1.12.0 (Blighe et al. 2021).

## Results

### Screening of bacterial strains

All of the seven screened bacterial strains transformed IBU, as determined by HPLC analysis (Table 2). Seven different transformation products, P1 to P7, were identified based on HPLC, LC-MS, GC-MS, and NMR analyses. All of the organisms produced P1, while none produced all seven transformation products.

**Table 2:** Biotransformation of ibuprofen and transformation products formed during the transformation of 0.005% ibuprofen by different bacterial strains. Shown are the relative decrease of IBU concentration and detected transformation products in the culture supernatant after 24 h of incubation of various bacterial strains with 0.005% IBU, cultivated with different carbon sources prior to incubation

Organism	Carbon source for cultivation	Ibuprofen decrease after 24 h (%)	Products detected after 24 h^1^					
			P1 + P2^2^	P3	P4	P5	P6	P7
*Priestia megaterium* SBUG 518	NB II LB	44.7 54.8	+++^2^ +++	++ ++	(+)^4^ (+)^4^	— —	—	—
*Priestia megaterium* SBUG 1979	NB II LB	3.4 2.3	+ +^2^	(+)^5^ —	— —	— —	—	—
*Bacillus pumilus* SBUG 1800	NB II	0	+^3^	—	—	—	—	—
*Bacillus thuringiensis* SBUG 1431	NB II	98.0	++++^3^	—	—	—	—	—
*Paenibacillus apiarius* SBUG 1947	NB II	0	+^2^	++	—	—	—	—
*Cupriavidus basilensis* SBUG 290	Biphenyl	0	+^2^	—	—	—	—	—
*Mycobacterium neoaurum* SBUG 109	Tetradecane	91.4	+++^2^	—	—	+	(+)^6^	(+)^6^

^1^ Product concentrations: +, ≤ 1 µg mL^−1^; ++, > 1 - ≤ 10 µg mL^−1^; +++, > 10 - ≤ 25 µg mL^−1^; ++++, > 25 µg mL^−1^; —, product not detected by HPLC analyses

^2^ Due to lack of base line separation of P1 and P2 during HPLC analyses, the products could not be quantified separately. Therefore, both products were quantified together using the 2-hydroxyibuprofen (P1) standard

^3^ Only P1 detected

^4^ P4 was only detected in incubation experiments supplemented with glucose; no standard for the calculation of its concentration was available

^5^ Detection not until 120 h of incubation (< 1 µg mL^-1^)

^6^ No standard for concentration calculation was available

*P. megaterium* SBUG 518 produced a high number and yield of transformation products, and was chosen as model organism for further studies.

### Structure elucidation of transformation products

Products formed during the incubation of the bacterial strains with IBU were identified by HPLC analyses via comparison of the UV-VIS spectrum and retention time with the data of authentic standards, as well as by GC-MS, LC-MS (Supp. Table S1) and/or NMR analyses (Table 3, Supp. Tables S2 to S6).

**Table 3:**
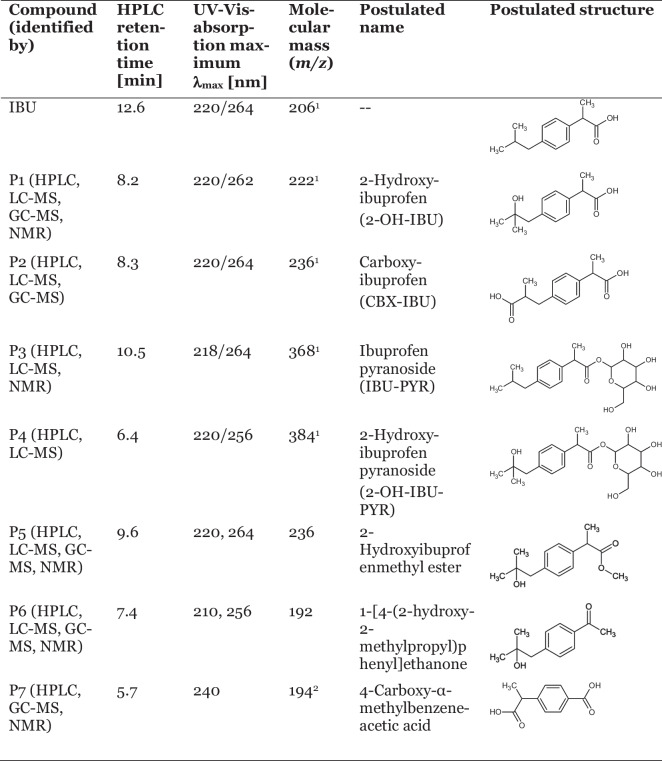
Transformation products formed during the transformation of IBU by various bacteria, identified by HPLC, LC-MS, GC-MS, and NMR analyses. Shown here are HPLC analysis results. For GC-MS and LC-MS results, see Supp. Table S1, for NMR results, see Supp. Tables S2 to S6. P1 to P4 were formed by *Priestia megaterium* SBUG 518 (and partially other strains studied) and will be discussed in more detail below. P5 to P7 were formed only by *Mycobacterium neoaurum* SBUG 109

^1^ determined by LC-MS analyses

^2^ determined by GC-MS analyses of the methylated compound

### Biotransformation experiments with *P. megaterium* SBUG 518

**Fig. 1:**
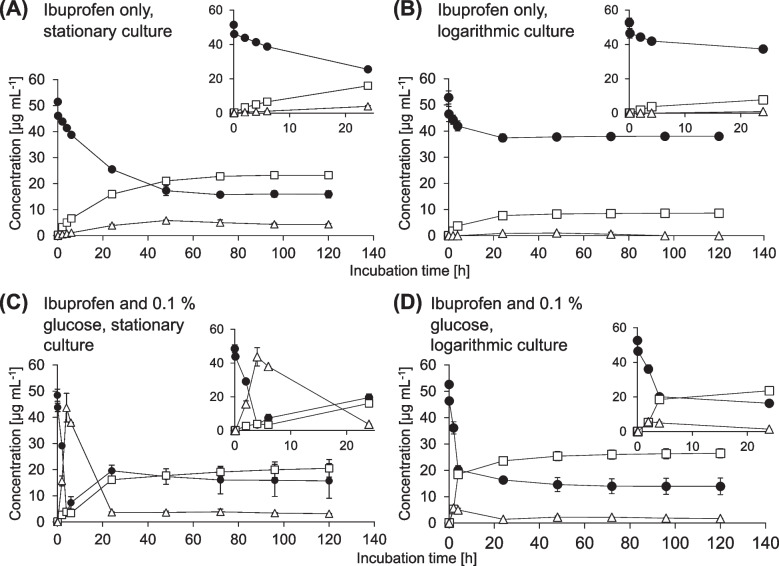
Time course of the biotransformation of 0.005 % ibuprofen (black circles) by *Priestia megaterium* SBUG 518 and formation of the transformation products 2-hydroxyibuprofen and carboxyibuprofen (squares), and ibuprofen pyranoside (triangles). Incubation was carried out with (**A**) stationary-phase cells, and (**B**) logarithmic-phase cells in the absence of glucose as well as with (**C**) stationary-phase cells, and (**D**) logarithmic-phase cells in the presence of 0.1% glucose. Small inserts show the first 24 h of incubation. Means and standard deviations of two independent parallels are shown

*Biotransformation of IBU in the absence of glucose leads to three main transformation products.* During 120 h of incubation of *P. megaterium* SBUG 518 with IBU, this strain transformed the drug to 2-hydroxyibuprofen (2-OH-IBU, product P1), carboxyibuprofen (CBX-IBU, P2), and ibuprofen pyranoside (IBU-PYR, P3), as well as to four more, unidentified products, one of which might be 2,3-dihydroxyibuprofen (data not shown). Transformation rate and amount of transformation products formed depended on the cellular growth phase: Stationary-phase cells transformed IBU to about 49% within 24 h (Figure 1A), while logarithmic-phase cells transformed about 29% within 24 h (Figure 1B). In both cases, the IBU concentration stagnated after these times. Using stationary-phase cells, all products accumulated in the culture supernatant (Figure 1A). For logarithmic-phase cells, 2-OH-IBU and CBX-IBU accumulated, while IBU-PYR was not detectable anymore after 96 h of incubation (Figure 1B).

**Fig. 2:**
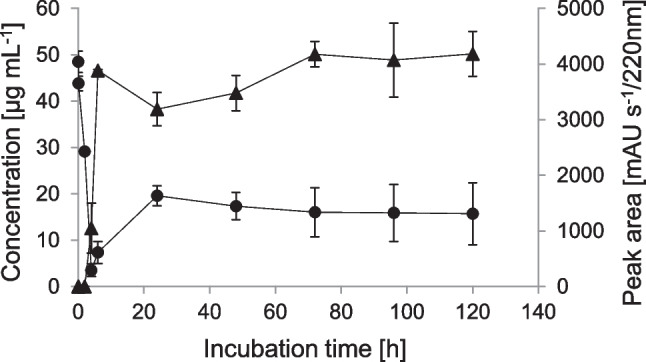
Time course of the biotransformation of ibuprofen (circles, primary axis) and the formation of 2-hydroxyibuprofen pyranoside (triangles, secondary axis) by stationary-phase cells of *Priestia megaterium* SBUG 518. Means and standard deviations of two independent parallels are shown

*Biotransformation of IBU in the presence of glucose yields higher concentrations of IBU-PYR.* After incubation of stationary-phase cells of *P. megaterium* SBUG 518 with IBU in the presence of 0.1% glucose, we detected the transformation product 2-OH-IBU-PYR (product P4) by HPLC analysis, in addition to 2-OH-IBU, CBX-IBU, and IBU-PYR. Stationary-phase cells transformed about 92% of IBU within the first 4 h of incubation, which was correlated with a sharp concentration increase of IBU-PYR (Figure 1C). After 4 h, IBU concentration increased again, whereas that of IBU-PYR decreased (Figure 1C). Thus, while about 49% of IBU was glycosylated after 4 h, after 24 h only about four percent of the drug remained glycosylated. The concentration of 2-OH-IBU-PYR increased in the same fashion as the IBU-PYR concentration, but with a 2 h-delay, and only slightly decreased in concentration after 6 h incubation (Figure 2).

Logarithmic-phase cells transformed about 73% IBU within 72 h to 2-OH-IBU, CBX-IBU, and IBU-PYR, with the highest transformation rate within the first 4 h of incubation (Figure 1D). All products accumulated in the culture supernatant until the end of incubation. The product 2-OH-IBU-PYR was detected only in one replicate after 4 h of incubation with logarithmic-phase cells.

*Cell cycle phase and glucose presence impact IBP biotransformation.* When no glucose was present in the medium, stationary-phase and logarithmic-phase cells transformed about the same amount of IBU within the first 4 h of incubation. However, at the end of the incubation, stationary-phase cells had transformed almost three times more IBU and formed triple the amount of 2-OH-IBU and CBX-IBU, as well as fourfold more IBU-PYR than logarithmic-phase cells.

Glucose presence increased transformation rates, especially in the first 4 h. Stationary-phase cells produced more IBU-PYR than logarithmic-phase cells, but glycosylation was reversible only in incubations with stationary-phase cells. After 24 h incubation with IBU and glucose, product quantities of cells in both growth phases were similar. Please refer to Table 4 for a summary of IBU biotransformation results. In addition, IBU apparently impacted sporulation of *P. megaterium* SBUG 518: logarithmic-phase cells with IBU (but without glucose) produced only few spores after 120 h incubation, whereas under all other three conditions mostly spores where present after 120 h incubation (Supp. Figure S1).

**Table 4:** Concentration of ibuprofen (IBU) and of products formed after 4 h, 24 h, and 120 h incubation with IBU of stationary-phase cells and logarithmic-phase cells, respectively, in the absence and presence of 0.1% glucose in the incubation medium. Conc.: concentration; P1: 2-hydroxyibuprofen; P2: carboxyibuprofen, P3: ibuprofen pyranoside, P4: 2-hydroxyibuprofen pyranoside. The “Sum” columns refer to the total concentration of ibuprofen contained in the quantifiable products P1, P2, and P3

		IBU and transformation products formed by											
		Stationary-phase cells						Logarithmic-phase cells					
		IBU		P1+P2	P3	P4	Sum IBU^1 ^(µg mL^‑1^)	IBU		P1+P2	P3	P4	Sum IBU^1 ^(µg mL^−1^)
Incubation conditions	Incubation time	Decrease of (%)	Conc. (µg mL^−1^)	Conc. (µg mL^‑1^)		Peak area (mAU s^‑1^/220 nm)		Decrease of (%)	Conc. (µg mL^−1^)	Conc. (µg mL^−1^)		Peak area (mAU s^‑1^/220 nm)	
Absence of glucose	4 h	10.2	41.4	5.0	0.8	0	46.5	9.8	41.9	3.8	0	0	45.4
	24 h	44.7	25.5	15.9	3.9	0	42.4	19.6	37.4	7.7	0.9	0	45.1
	120 h	65.4	15.9	23.2	4.3	0	39.8	18.2	38.0	8.6	0	0	46.0
Presence of 0.1% glucose	4 h	91.9	3.5	3.9	43.7	1050	31.6	56.6	20.2	18.5	5.0	0	40.2
	24 h	55.4	19.6	16.1	3.8	3187.5	36.7	64.8	16.3	23.6	1.4	105.8	39.0
	120 h	64.4	15.7	20.5	3.2	4181.7	36.5	70.0	13.9	26.5	1.7	0	39.4

^1^ As P1 and P2 were not base line separated during HPLC analyses, both products were quantified together; and the molar mass of 2-hydroxyibuprofen was used for mass balance calulation

**Fig. 3:**
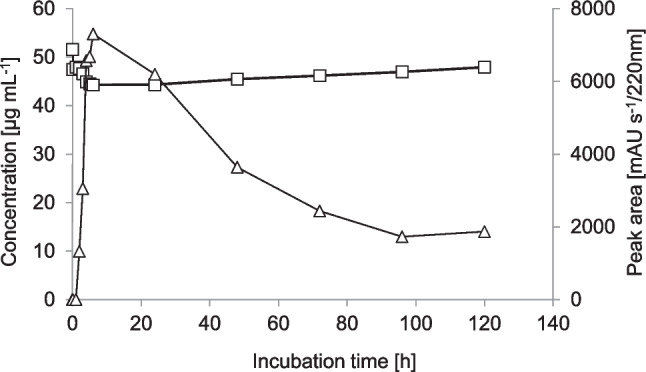
Time course of the biotransformation of 2-hydroxyibuprofen (squares) and the formation of 2-hydroxyibuprofen pyranoside (triangles) by *Priestia megaterium* SBUG 518. This experiment was carried out once

*2-OH-IBU is glycosylated in the presence of glucose.* When using 2-OH-IBU as biotransformation substrate in presence of glucose, stationary-phase cells formed 2-OH-IBU-PYR (product P4) with a very low transformation rate. Only about 7% of 2-OH-IBU was transformed by the cells within the first 6 h, during which time product concentration increased. Thereafter, product concentration decreased and substrate concentration increased (Figure 3).

**Fig. 4:**
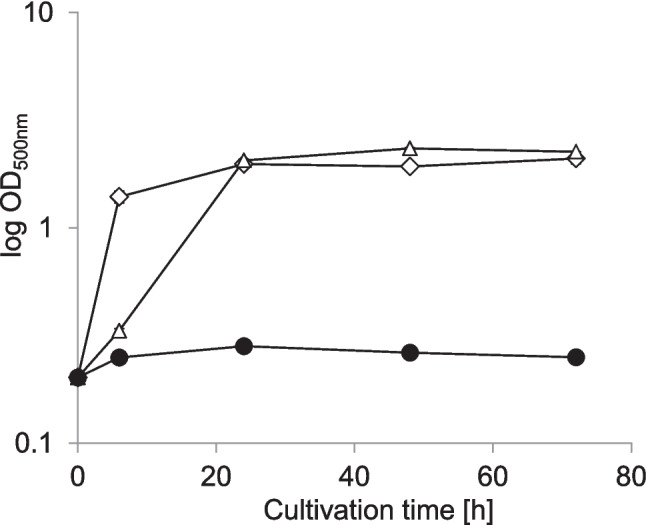
Effect of 0.05% (2.19 mM) ibuprofen (black circles) and the equimolar concentration of the transformation product ibuprofen pyranoside (triangles) on the growth of *Priestia megaterium* SBUG 518 in NBII (pH 6.0) compared to the control (diamonds) without ibuprofen or transformation product. Shown are means and standard deviations of two independent parallels

*The transformation product IBU-PYR is less toxic than the drug IBU.* IBU inhibited the growth of *P. megaterium* SBUG 518 at a concentration of 0.05% (equivalent to 2.19 mM) in NB II, pH 6.0 (but not in NB II with un-adjusted pH of 7.2, data not shown). However, with the transformation product IBU-PYR (P3) in equimolar concentration, growth of *P. megaterium* SBUG 518 was only slightly delayed compared to the control (Figure 4).

**Fig. 5:**
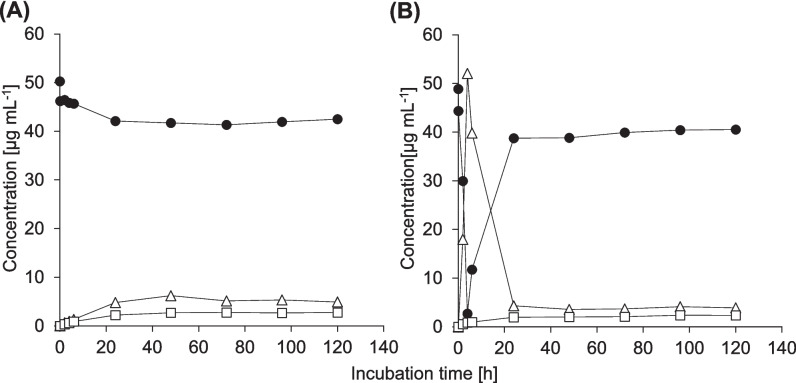
Time course of the biotransformation of ibuprofen (black circles) by *Priestia megaterium* SBUG 518 and formation of the transformation products 2-hydroxyibuprofen (squares) and ibuprofen pyranoside (triangles) in the presence of (**A**) 219 μM 1‑aminobenzotriazole and of (**B**) 219 μM 1‑aminobenzotriazole and 0.1% glucose. These experiments were performed once. Please refer to Figure 1A and 1 C for the respective transformations without 1-aminobenzotriazole

*Cytochrome P450 inhibition leads to decreased IBU oxidation.* In the presence of the cytochrome P450 inhibitor 1-aminobenzotriazole, CBX-IBU and only about a tenth of the concentration of 2-OH-IBU as compared to the control without inhibitor was formed (Figure 5A). In contrast, the inhibitor had no effect on IBU-PYR concentrations.

Almost the same results were obtained in the presence of 1-aminobenzotriazole and 0.1% glucose. Only about one tenth of the concentration of 2-OH-IBU compared to control conditions, and neither CBX-IBU nor 2-OH-IBU-PYR were detected, whereas reversible formation of IBU-PYR occurred (Figure 5B).

### Influence of IBU on the proteome profile of P. megaterium SBUG 518

**Fig. 6:**
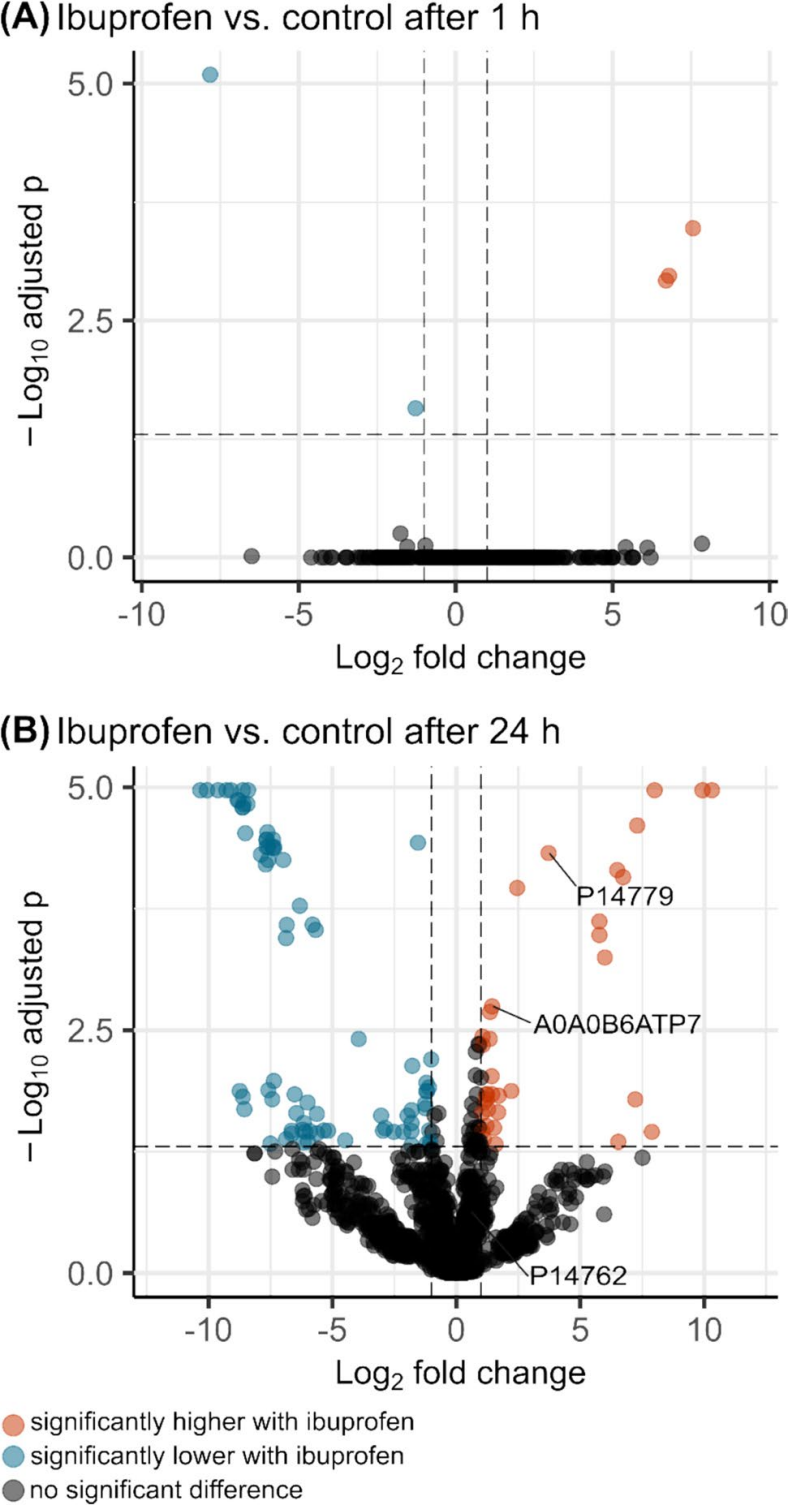
Volcano plots showing differences in the proteome of *Priestia megaterium* SBUG 518 after incubation with ibuprofen as compared to a control without ibuprofen. **A** Proteome comparison after 1 h incubation, **B** proteome comparison after 24 h incubation. Significance: FDR < 0.05, log_2_ fold change ≥ 1/≤− 1. Cytochrome P450 proteins are marked (A0A0B6ATP7: Cytochrome P450 family protein, P14779: bifunctional cytochrome P450/NADPH-P450 reductase Cyp102A1 (P450 BM3), P14762: Cytochrome P450 BM1)

In total, we identified 1,346 *P. megaterium* SBUG 518 proteins after 1 and 24 h incubation of stationary-phase cells with vs. without IBU (Supp. Table S7). Of these, after 1 h two secretion family proteins and one sporulation protein were significantly higher abundant in the IBU incubations as compared to the respective controls (Figure 6, Supp. Table S8). Proteins with significantly lower abundance in the IBU incubations after 1 h included a spore protein and an isocitrate lyase (Supp. Table S7). After 24 h, in total 36 proteins were significantly higher abundant in the IBU-incubated cells. These included two cytochrome P450 proteins, one of them being the bifunctional cytochrome P450/NADPH-P450 reductase Cyp102 A1 (P14779, also known as P450 BM3), as well as iron-containing alcohol dehydrogenases (FeADHs), aldehyde dehydrogenases, and acetyl-CoA dehydrogenases. Moreover, proteins of the amino acid metabolism, and two lipid/propionate metabolism enzymes, 3-hydroxypropionyl-coenzym A dehydratase and enoyl-CoA hydratase/isomerase, as well as the relaxosome subunit MobC where higher abundant in IBU incubations after 24 h.

In addition, we detected two HlyD family transporters, as well as single-stranded DNA-specific exonuclease only after incubation of *P. megaterium* SBUG 518 with IBU (Supp. Table S8). After 24 h, in total 81 proteins were significantly lower abundant in the IBU incubations as compared to the control without IBU. These included the fatty acid biosynthesis enzymes malonyl CoA-acyl carrier protein transacylase, enoyl-[acyl-carrier-protein] reductase, the oxidative stress proteins catalase and alkyl hydroperoxide reductase, as well as ribosomal proteins, and proteins involved in sporulation, amino acid biosynthesis, and stress response proteins (Supp. Table S7).

## Discussion

We here show that *P. megaterium* SBUG 518 has high potential for IBU transformation, but cannot use IBU as carbon source. *P. megaterium* SBUG 518 exhibited two general IBU biotransformation pathways, which we will discuss in detail below: (A) isobutyl side chain hydroxylation at two positions, and (B) conjugation with a sugar molecule. Conjugation with sugar took place almost exclusively in presence of glucose, which can be expected. More surprisingly, stationary-phase cells exhibited higher transformation rates as compared to logarithmic-phase cells.

(A) Isobutyl side chain hydroxylation yielded the products 2-OH-IBU, which accumulated in high concentrations, and CBX-IBU. While 2-OH-IBU generation by bacteria has hitherto seldomly be demonstrated (Marchlewicz et al. 2017b), it is the main metabolite in urine of humans after IBU uptake (Kepp et al. 1997; Magiera and Gülmez 2014), and also a major transformation product of other higher organisms like animals (Gomez et al. 2011; Waraksa et al. 2018), plants (Li et al. 2016), and fungi (Marco-Urrea et al. 2009; Borges et al. 2011). Consequently, 2-OH-IBU is widespread in the environment. It can be detected in WWTPs (Ferrando-Climent et al. 2012; Kim et al. 2014), in river water and river sediments (Löffler and Ternes 2003; Dai et al. 2018), and in other aquatic environments (de García et al. 2013). While CBX-IBU appears to be an unusual IBU transformation product in bacteria and fungi, it is produced by humans, some animals (Kepp et al. 1997; Magiera and Gülmez 2014; Waraksa et al. 2018), and plants (Li et al. 2016). In the environment, CBX-IBU is present in wastewater, activated sludge, and river biofilm reactors (Buser et al. 1999; Winkler et al. 2001; Ferrando-Climent et al. 2012). Despite that CBX-IBU is formed only after initial oxidation of IBU to 3-OH-IBU, we did not detect the primary oxidation product 3-OH-IBU, likely because the further oxidation to CBX-IBU happened too fast. Likewise, 3-OH-IBU is not, or at lower concentrations than CBX-IBU, detected in urine and environmental samples (Kepp et al. 1997; Buser et al. 1999; Winkler et al. 2001; Waraksa et al. 2018). While we did not find additional IBU hydroxylation products in our study with *P. megaterium* SBUG 518, other organisms also produce 1-OH-IBU (*Phanerochaete chrysosporium*, Rodarte-Morales et al. 2012) and 1,2-diOH-IBU (*Trametes versicolor*, Marco-Urrea et al. 2009). According to our proteomics results, the two significantly higher abundant cytochrome P450 proteins in presence of IBU are likely the primary site of IBU hydroxylation in *P. megaterium* SBUG 518. This is corroborated by our cytochrome P450 inhibition assay results. Cytochrome P450 BM3, which we detected in over 12-fold higher abundance after 24 h IBU incubation as compared to the control, is one of two different described cytochrome P450 systems in *P. megaterium* (Sariaslani 1991; Warman et al. 2005). It consists of a P450 fatty acid hydroxylase and a mammal-like, albeit soluble, diflavin NADPH-P450 reductase in a single enzyme (Munro et al. 2002). P450 BM3 can be induced by IBU (English et al. 1996), and this enzyme can transform a variety of drugs to different metabolites (Di Nardo and Gilardi 2012). For example, in *P. megaterium* ATCC 14581, P450 BM3 oxidizes fatty acids, long-chain alcohols, and amides (Miura and Fulco 1975; Black et al. 1994). For further details on proteins potentially involved in IBU transformation to carboxyibuprofen, please refer to the Supplementary Discussion.

(B) Conjugation with a sugar molecule, or glycosylation, was the main driver of the high IBU transformation rate of *P. megaterium* SBUG 518 with up to 90% transformation in 4 h. IBU was conjugated with a pyranose, yielding a glucoside. Given that this transformation took place almost exclusively in presence of glucose, the pyranose is likely to be glucose. The carboxylic group of IBU enables direct conjugate formation with the parent substrate. This is in contrast to the metabolism of many other xenobiotics, where a functional group for conjugation has to be introduced in the so-called phase I metabolism, and only then a conjugate is formed via this new part of the molecule in phase II metabolism (Gonzalez and Tukey 2006). We postulate that the glycosylation of 2-OH-IBU, which we demonstrated in addition to that of IBU itself, also happens via the already-present carboxylic group, and not via the newly introduced hydroxyl group, and therefore does not adhere to “classical” xenobiotic metabolism. While IBU conjugation appears to be rare in bacteria, it is an important IBU metabolism pathway in humans, where foremost esterification via the carboxylic group of IBU and the hydroxylic group of glucuronic acid takes place, leading to acyl glucuronides (Kepp et al. 1997). In addition, in human liver microsomes IBU is conjugated to glucose, and human phase I metabolites might be glycosylated, too (Buchheit et al. 2011). The conjugation was highly reversible: potentially, the sugar is cleaved and used as energy source by the bacteria upon depletion of external and internal energy reserves.

We did not observe IBU mineralization by *P. megaterium* SBUG 518. Mineralization by other bacteria includes ligation to CoA or acidic side chain removal and ring cleavage, as well as co-metabolic degradation, and IBU can even be used as carbon source (Murdoch and Hay 2005, 2013, 2015; Almeida et al. 2013; Marchlewicz et al. 2017b, 2017a; Aguilar-Romero et al. 2021; Ivshina et al. 2021).

**Fig. 7:**
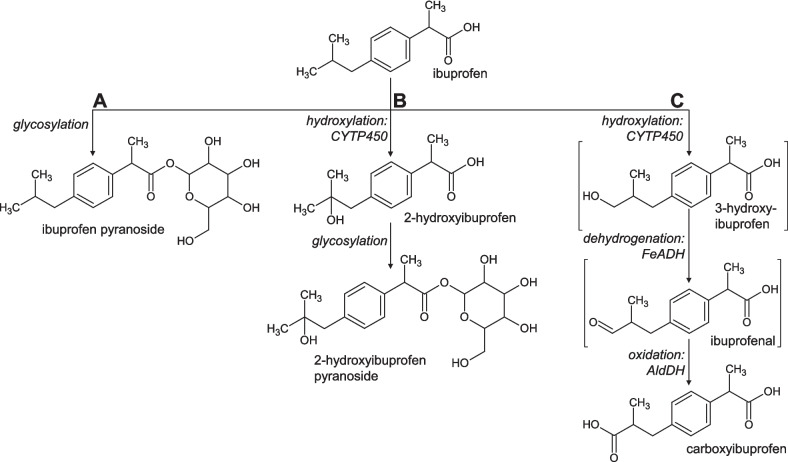
Proposed transformation pathways of ibuprofen by *Priestia megaterium* SBUG 518 to (**A**) ibuprofen pyranoside, (**B**) 2-hydroxyibuprofen pyranoside, and (**C**) carboxyibuprofen. Products in square brackets are proposed intermediates, which were not detected in this study. Italics: transformation reaction and proposed enzymes according to our proteomics analysis (no enzyme for glycosylation could be proposed, as transformation experiments for proteomics did not contain glucose). CYTP450: cytochrome P450, FeADH: iron-containing alcohol dehydrogenase, AldDH: aldehyde dehydrogenase

In summary, we propose three different pathways of IBU metabolism in *P. megaterium* SBUG 518 (Figure 7).

Based on our results, conjugation of IBU likely serves as an efficient detoxification mechanism in *P. megaterium* SBUG 518, as the IBU-PYR exhibited no toxic effect on growth of *P. megaterium* SBUG 518 in equimolar concentration to almost completely growth-abolishing IBU concentrations. Our proteomic analyses enabled us to shed light on the molecular basis for IBU transformation and IBU toxicity in *P. megaterium* SBUG 518. The detected HlyD transporters likely transport IBU and its transformation products out of the cells: HlyD family transporters belong to ABC transporters, which in Gram-positive bacteria are often used to expel xenobiotics (Lubelski et al. 2007). Apparently, IBU also elicits a systemic response of *P. megaterium* SBUG 518. Especially sporulation seems to be impacted by the drug, but also DNA damage is indicated, and an impact on amino acid metabolism and protein synthesis. For details, please refer to the Supplementary Discussion.

## Conclusion and outlook

*P. megaterium* SBUG 518 exhibits IBU transformation and detoxification mechanisms similar to the respective cytochrome P450-dependent transformation in humans. Therefore, *P. megaterium* SBUG 518 seems to be well suited as a model organism for further research of drug metabolism, not least because its cytochrome P450 monooxygenase itself also highly resembles the corresponding eukaryotic enzymes.

The reversibility of the fast glycosylation of IBU can have practical consequences: It is an open question why, despite the apparently nearly complete removal of IBU in WWTPs, it can still be detected in various surface waters. A temporary “masking” of IBU as IBU-PYR could explain this. Therefore, kinetic data of IBU transformation should take into account possible conjugate formation.

Future studies prompted by our research should include incubation of *P. megaterium* SBUG 518 with environmentally relevant ibuprofen concentrations, as well as of organisms with ibuprofen in presence of glucose, with both ibuprofen enantiomers separately, and with other ibuprofen transformation products than IBU-PYR, to elucidate toxicity and transformation pathways in more detail. Additionally, contrasting the proteome of logarithmic-phase cells when incubated with IBU with that of stationary-phase cells presented here would allow more insights into the comparatively lower transformation capacity of logarithmic-phase cells. Heterologous expression and gene knockout experiments would provide means of proving our proposed IBU transformation pathways in *P. megaterium* SBUG 518.

## Supplementary Information

Below is the link to the electronic supplementary material.

Supplementary Material 1 (DOCX 922 KB)

Supplementary Material 1 (XLSX 370 KB)

## Data Availability

The mass spectrometry proteomics data have been deposited to the ProteomeXchange Consortium via the PRIDE (Perez-Riverol et al. 2022) partner repository with the dataset identifier PXD015716.
