# Supplementary material for: Transformation of the drug ibuprofen by Priestia megaterium: reversible glycosylation and generation of hydroxylated metabolites
Source: Environ Sci Pollut Res Int. 2025 Apr 21;32(19):11981–95. doi: 10.1007/s11356-025-36393-5 (PMC12049389; doi:10.1007/s11356-025-36393-5)
Supplement: Supplementary file 1 — Supplementary Material 1 (DOCX 922 KB) [file 11356_2025_36393_MOESM1_ESM.docx]

**Supplementary Information**

**Transformation of the drug ibuprofen by *Priestia megaterium*: Reversible glycosylation and generation of hydroxylated metabolites**

Tjorven Hinzke^1,2^, Rabea Schlüter^1^, Annett Mikolasch^1^, Daniela Zühlke^1^, Patrick Müller^1^, Robert Kleditz^1^, Katharina Riedel^1^, Michael Lalk^3^, Dörte Becher^1^, Halah Sheikhany^1§^, Frieder Schauer^1§^

^1^ Institute of Microbiology, University of Greifswald, Felix-Hausdorff-Straße 8, 17489 Greifswald, Germany

^2^ Helmholtz Institute for One Health, Helmholtz-Centre for Infection Research (HZI), 17489 Greifswald, Germany

^3^ Institute of Biochemistry, University of Greifswald, Felix-Hausdorff-Straße 4, 17489 Greifswald, Germany

^§^ deceased.

Corresponding author: Tjorven Hinzke. e‑mail: tjorven.hinzke@uni-greifswald.de

# Supplementary Methods

## GC–MS

The metabolites were detected and quantified by injecting 1 µL of the extract into an Agilent gas chromatograph 7890A GC System (Waldbronn, Germany) equipped with a capillary column (Agilent 1901 S-433, 30 m x250 µm x 0.25 µm, HP-5ms column) and a mass selective detector 5975C inert XL EI/CI MSD with a quadrupole mass spectrometer. The injector was operated at 250°C and the injection was pulsed splitless at 10.5 psi for 2 min. For the analysis of extracts we used an elution profile: the column temperature started at 80 °C for 5 min and increased to 280 °C at 10 K min^-1^ and was finally maintained at 280 °C for 10 min. To analyze the acid-extractable products P1, P2 and P7, the extracts after acidic extraction at pH 2 were derivatized by methylation with diazomethane as described by (De Boer and Backer, 1956) using a micro-apparatus (Aldrich-Chemie, Steinheim, Germany). All products with carboxyl groups were methylated, one methyl group per carboxyl group. The mass spectrometer conditions were set to 250°C for the interface, the source conditions were 230°C and the quadrupole temperature was set to 150°C.

## Proteome analysis of Priestia megaterium SBUG 518 during incubation with ibuprofen

For cell lysis of *P. megaterium* SBUG 518, 500 µL glass beads (diameter 0.10–0.11 mm, Sartorius AG, Göttingen, Germany) were added to 1 mL sample and cells disrupted with a FastPrep®24 homogenizer (M. P. Biomedicals, Irvine, Cali­fornia, USA) in three 30 s cycles at 6.5 m s^‑1^ with cooling samples on ice for 5 min between cycles. To remove glass beads and cell debris, samples were centrifuged twice (5 min, 21,885 x *g*, 4 °C, Heraeus Biofuge Primo R) and the supernatant transferred to a new tube. Proteins were precipitated overnight with six times the sample volume of ice-cold acetone at ‑20 °C. Subsequently, we pelleted proteins by centrifugation for 1 h at 10,015 x g and 4 °C, discarded the supernatant and resuspended the protein pellet in 1 mL ice-cold acetone. After repeating the washing step, we dried the protein pellets at room temperature and then resuspended them in 0.5 mL 8 M urea/ 2 M thiourea. Following centrifugation for 10 min at 10,015 x g and room temperature, the supernatant was transferred into a new tube. Protein concentration was determined with Roti^®^-Nanoquant (Roth, Karlsruhe, Germany) reagent according to the manufacturer’s instructions. Protein solutions were stored at -20 °C. Experiments were performed in triplicate. Proteins were separated using 1D-SDS-PAGE as described in Zühlke et al. (2017), using precast 4-20 % polyacrylamide gels (4‑20 % Criterion^TM^ TGX BioRad^TM^, BIO-RAD, Hercules, USA).

Subsequent LC-MS-MS (LC: nanoACQUITY^TM^-UPLC^TM^-System (Waters, Milford, USA); MS-MS: linear trap quadrupole (LTQ)-Orbitrap mass spectrometer (Thermo Fisher Scientific, Waltham, USA)) analysis was done as follows: Peptides were loaded on a pre-column (Symmetry C18, 5 μm, 180 μm inner diameter x 20 mm, Waters, Milford, USA) and washed for 3 min at a flow rate of 10 μL min^-1^ with 0.1 % acetic acid. Peptides were then eluted onto the analytical column (BEH130 C18, 1.7 μm, 100 μm inner diameter x 100 mm, Waters, Milford, USA) with a step-wise gradient of 5 to 99% acetonitrile in 0.1% acetic acid in 80 min at a flow rate of 400 nL min^-1^ (0.5% acetonitrile min^-1^ to 25% acetonitrile, 0.83% acetonitrile min^-1^ to 50% acetonitrile, 49% acetonitrile min^-1^ to 99% acetonitrile in 0.1% acetic acid). Measurements were performed in LTQ/Orbitrap parallel mode. Survey scans were performed in the orbitrap (m/z range 300–2,000, resolution 30,000, lock mass option enabled, lock mass 445.120025). Per scan, the five peaks with highest intensity were fragmented in the LTQ using collision induced dissociation (CID). Precursor ions with unknown as well as those with single charge were excluded from fragmentation. Dynamic exclusion for precursor ions was set to 30 s.

# Supplementary Results

Table S1: GC-MS and LC-MS data of ibuprofen and its analogues formed during the incubation of *Priestia megaterium* SBUG 518 with ibuprofen. Chemical structures are given in Table 2 in the main text.

| Product | Theoretical molecular weight  (g mol^-1^) | Mass difference between substrate and transformation product | LC-MS data | | Base peak ion or fragment ions (GC-MS) |
| --- | --- | --- | --- | --- | --- |
|  |  |  | negative ion mode | positive ion  mode [M+H]^+^ |  |
| Substrate ibuprofen (molecular weight 206 g mol^-1^) | | | | | |
| P1 (2-hydroxyibuprofen) | 222 | ∆ 16 g mol^-1^ | *m/z* 221 [M-H]^-^ | *m/z* 240 [M+NH_4_^+^]^+^ | P1 *m/z* 59, 91, 118, 119, 178  (fragment m/z 178 corresponds to a cleavage at the hydroxylated aliphatic side chain of m/z 44)  St^1^*m/z* 59, 91, 118, 119, 178 |
| P2 (carboxyibuprofen) | 236 | ∆ 30 g mol^-1^ | *n.d.* | *m/z* 254 [M+NH_4_^+^]^+^ | *m/z* 264 (M^+^+2CH_3_)^2^ |
| P3 (ibuprofen pyranoside) | 368 | ∆ 162 g mol^-1^ | *m/z* 413 [M+HCOO^-^]^-^  *m/z* 367 [M-H]^-^ | *m/z* 386 [M+NH_4_^+^]^+^ | n.d. |
| P5 (2-hydroxyibuprofen-methyl ester) | 236 |  | *n.d.* | *m/z* 254 [M+NH_4_^+^]^+^ | *m/z* 59, 91, 118, 119, 178  MS spectrum identical with MS spectrum of P1 |
| P6 (1-[4-(2-hydroxy-2-methyl-propyl)phenyl]ethanone ) | 192 |  | *n.d.* | *m/z* 193 [M+H^+^]^+^ | m/z 43, 59, 91, 134, 177 |
| P7 (4-Carboxy-α-methylbenzeneacetic acid) | 194 | ∆ 12 g mol^-1^ | n.d. | n.d. | *m/z* 222 (M^+^+2CH_3_) |
| Substrate 2-hydroxy-ibuprofen (P1, molecular weight 222 g mol^-1^) | | | | | |
| P4 | 384 | ∆ 162 g mol^-1^ | *m/z* 429 [M+HCOO^-^]^-^  *m/z* 383 [M-H]^-^ | *m/z* 402 [M+NH_4_^+^]^+^ | n.d. |

n.d. not detected; ^1^ Bought standard compound 2-hydroxyibuprofen and product P1 have the same MS spectra. ^2^ M^+^+2CH_3_ dimethylated-derivative according to two carboxyl groups per molecule.

Table S2: NMR data and structure of 2-hydroxyibuprofen (product P1) formed during the incubation *Priestia megaterium* SBUG 518 with ibuprofen.

| ^1^H  Chemical shift^1^ (multiplicity, coupling constant, number of protons, proton assignment) | ^1^H-^13^C-correlation | ^13^C  Chemical shift^1^ (assignment of carbon atoms) | structure |
| --- | --- | --- | --- |
| 1.06 (s, 6H, H-12, H-13) | 29.2 (C-12, C-13), 49.0 (C-10), 69.3 (C-11) | 29.2 (C-12, C-13) | 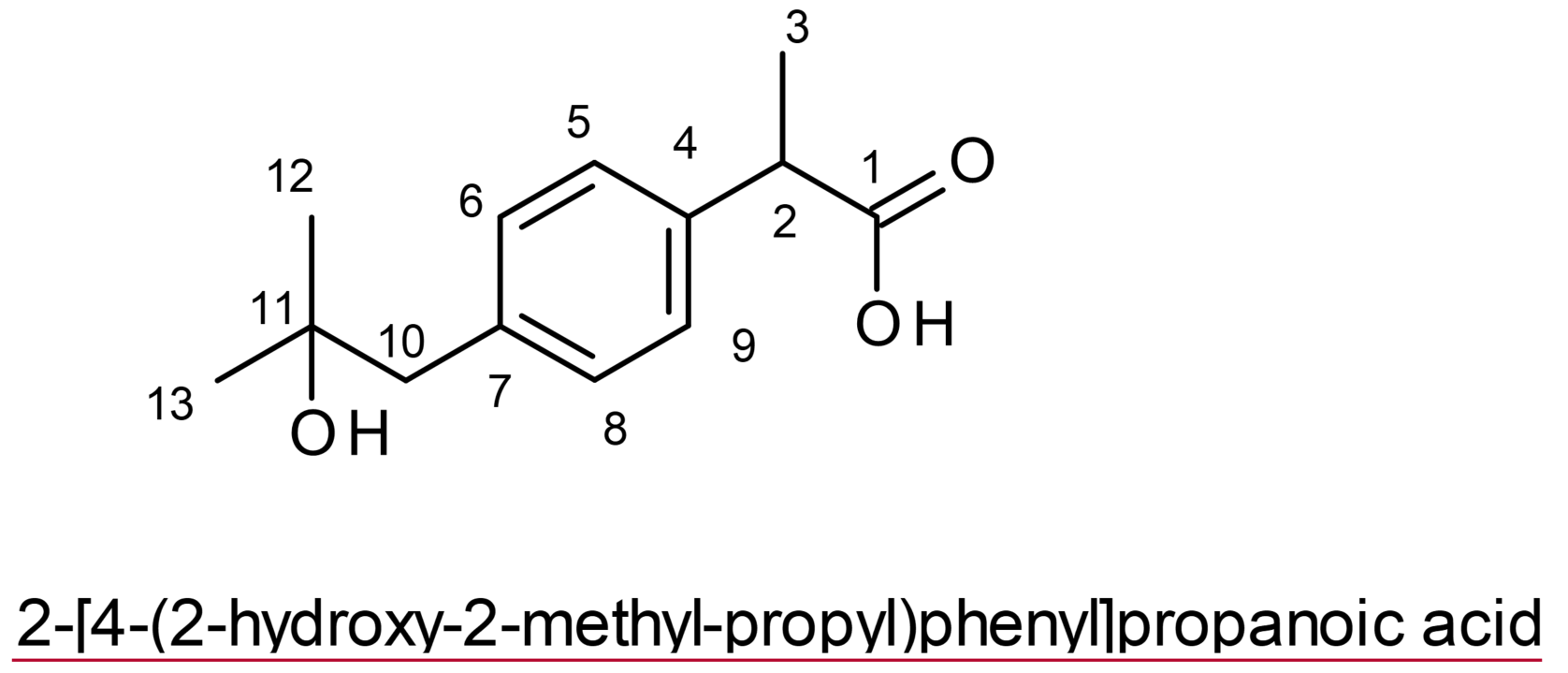 |
| 1.35 (d, J = 7.1 Hz, 3H, H-3) | 44.3 (C-2), 138.5 (C-4), 175.4 (C-1) | 18.5 (C-3) |  |
| 2.62 (s, 2H, H-10) | 29.2 (C-12, C-13), 69.3 (C-11), 130.4 (C-6, C-8), 137.3 (C-7) | 49.0 (C-10) |  |
| 3.62 (m, J = 7.1 Hz, 1H, H-2) | 18.5 (C-3), 126.5 (C-5, C-9), 138.5 (C-4), 175.4 (C-1) | 44.3 (C-2) |  |
| 7.15 (d, J = 8.3 Hz, 2H, H-6, H-8) | 49.0 (C-10), 130.4 (C-6, C-8), 138.5 (C-4) | 130.4 (C-6, C-8) |  |
| 7.17 (d, J = 8.3 Hz, 2H, H-5, H-9) | 44.3 (C-2), 126.5 (C-5, C-9), 137.3 (C-7) | 126.5 (C-5, C-9) |  |
|  |  | 69.3 (C-11) |  |
|  |  | 137.3 (C-7) |  |
|  |  | 138.5 (C-4) |  |
|  |  | 175.4 (C-1) |  |

^1^ Chemical shifts are expressed in d (ppm) calibrated on the resonances of the residual nondeuterated solvent DMSO.

Table S3: NMR data and structure of ibuprofen pyranoside (product P3) formed during the incubation of *Priestia megaterium* SBUG 518 with ibuprofen.

| ^1^H  Chemical shift^1^ (multiplicity, coupling constant, number of protons, proton assignment) | ^1^H-^13^C-correlation | ^13^C  Chemical shift^1^ (assignment of carbon atoms) | structure |
| --- | --- | --- | --- |
| 0.87 (m, J = 6.6 Hz, 6H, H-12, H-13) | 22.7 (C-12, C-13), 30.0 (C-11), 44.7 (C-10) | 22.7 (C-12, C-13) |  |
| 1.42 (d, J = 7.2 Hz, 3H, H-3) | 44.63, 44.67 (C-2), 137.76, 137.77 (C-4),173.3 (C-1) | 19.1, 19.5 (C-3) |  |
| 1.83 (m, J = 6.7 Hz, 1H, H-11) | 22.7 (C-12, C-13), 44.7 (C-10), 140.20, 140.32 (C-7) | 30.0 (C-11) |  |
| 2.43 (d, J = 7.2 Hz, 2H, H-10) | 22.7 (C-12, C-13), 30.0 (C-11), 129.4, 129.5 (C-6, C-8), 140.20, 140.32 (C-7) | 44.7 (C-10) |  |
| 3.12 (m, J = 9.0 Hz, 2H, H-pyr*^2^*) | (61.02, 61.06 (C6’)), 78.4 (C-pyr), 95.2, 95.3 (C-pyr) | 70.0 (C-pyr), 72.09, 73.03 (C-pyr) |  |
| 3.20 (m, 1H, H-pyr) | 95.2, 95.3 (C-pyr) | 78.4 (C-pyr) |  |
| 3.23 (m, J = 9.0 Hz, 1H, H-pyr) | 70.0 (C-pyr) | 77.0 , 77.1 (C-pyr) |  |
| 3.45 (m, 1H, H-6’) | - | 61.02, 61.06 (C-6’) |  |
| 3.64 (m, J = 11.8 Hz, J = 12.2 Hz, J = 12.8 Hz, 1H, H-6’) | - | 61.02, 61.06 (C-6’) |  |
| 3.79 (m, J = 7.2 Hz, 1H, H-2) | 19.1, 19.5 (C-3), 127.7, 127.8 (C-5, C-9), 137.76, 137.77 (C-4), 173.3 (C-1) | 44.63, 44.67 (C-2) |  |
| 5.36 (m, J = 8.2 Hz, J = 1.8 Hz, 1H, H-1’) | 72.09, 73.03 (C-pyr), 77.0, 77.1 (C-pyr), 78.4 (C-pyr), 173.3 (C-1) | 95.2, 95.3 (C-1’) |  |
| 7.12 (m, J = 8.1 Hz, J = 3.9 Hz, 2H, H-6, H-8) | 44.7 (C-10), 129.4, 129.5 (C-6, C-8), 137.76, 137.77 (C-4) | 129.4, 129.5 (C-6, C-8) |  |
| 7.23 (m, J = 8.1 Hz, J = 3.9 Hz, 2H, H-5, H-9) | 44.63, 44.67 (C-2), 127.7, 127.8 (C-5, C-9), 140.20, 140.32 (C-7) | 127.7, 127.8 (C-5, C-9) |  |
|  |  | 137.77 (C-4) |  |
|  |  | 140.32 (C-7) |  |
|  |  | 173.3 (C-1) |  |

^1^ Chemical shifts are expressed in d (ppm) calibrated on the resonances of the residual nondeuterated solvent DMSO.

^2^ Pyr corresponds to protons and carbon atoms of the pyranoside part (1’ to 6’) of the structure which cannot be determined exactly.

Table S4: NMR data and structure of 2-hydroxyibuprofen methyl ester (product P5) formed during the incubation of *Mycobacterium neoaurum* SBUG 109 with ibuprofen.

| ^1^H  Chemical shift^1^ (multiplicity, coupling constant, number of protons, proton assignment) | ^1^H-^13^C-correlation | ^13^C  Chemical shift^1^ (assignment of carbon atoms) | structure |
| --- | --- | --- | --- |
| 1.06 (s, 6H, H-12, H-13) | 29.7 (C-12, C-13), 49.5 (C-10), 69.8 (C-11), (138.1 (C-7)) | 29.7 (C-12, C-13) | 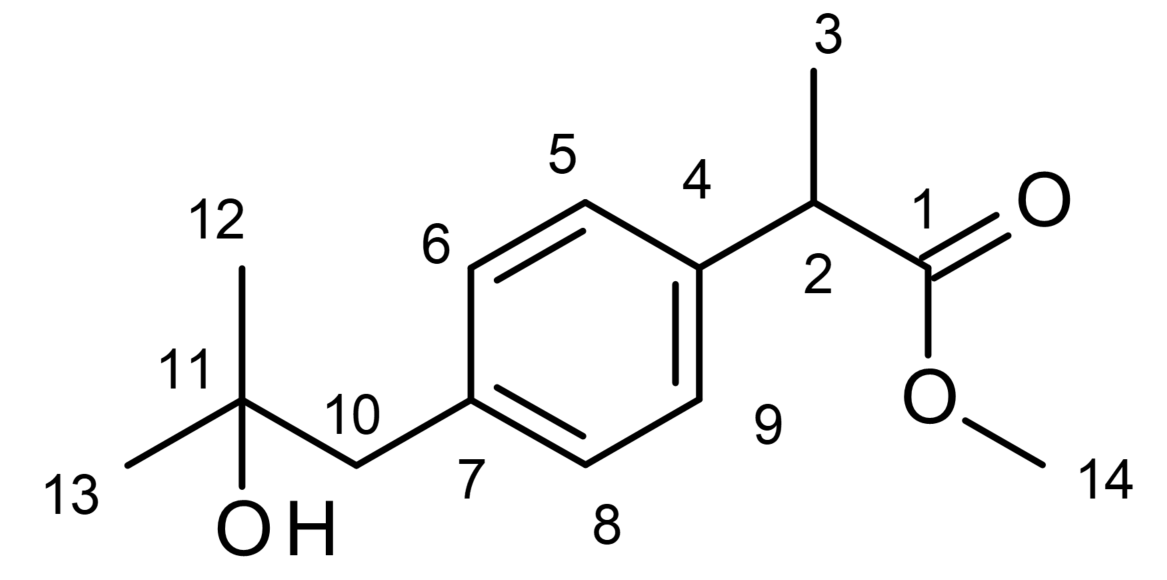 |
| 1.39 (d, J = 7.1 Hz, 3H, H-3) | 44.6 (C-2), 138.4 (C-4), 175.9 (C-1) | 19.0 (C-3) |  |
| 2.63 (s, 2H, H-10) | 29.7 (C-12, C-13), 69.8 (C-11), 131.0 (C-6, C-8), 138.1 (C-7) | 49.5 (C-10) |  |
| 3.60 (s, 3H, H-14) | 175.9 (C-1) | 52.1 (C-14) |  |
| 3.76 (m, J = 7.1 Hz, 1H, H-2) | 19.0 (C-3), 127.0 (C-5, C-9), 138.4 (C-4), 175.9 (C-1) | 44.6 (C-2) |  |
| 7.17 (s, 4H, H-5, H-6, H-8, H-9) | 44.3 (C-2), 49.5 (C-10), 127.0 (C-5, C-9), 131.0 (C-6, C-8), 138.1 (C-7), 138.4 (C-4) | 127.0 (C-5, C-9), 131.0 (C-6, C-8) |  |
|  |  | 69.8 (C-11) |  |
|  |  | 138.1 (C-7) |  |
|  |  | 138.4 (C-4) |  |
|  |  | 175.9 (C-1) |  |

^1^ Chemical shifts are expressed in d (ppm) calibrated on the resonances of the residual nondeuterated solvent DMSO.

Table S5: NMR data and structure of 1-[4-(2-hydroxy-2-methylpropyl)phenyl]ethanone (product P6) formed during the incubation of *Mycobacterium neoaurum* SBUG 109 with ibuprofen.

| ^1^H  Chemical shift^1^ (multiplicity, coupling constant, number of protons, proton assignment) | ^1^H-^13^C-correlation | ^13^C  Chemical shift^1^ (assignment of carbon atoms) | structure |
| --- | --- | --- | --- |
| 1.08 (s, 6H, H-, H-11, H-12) | 29.7 (C-11, C-12), 49.7 (C-9), 69.8 (C-10), (145.3 (C-6)) | 29.7 (C-11, C-12) | 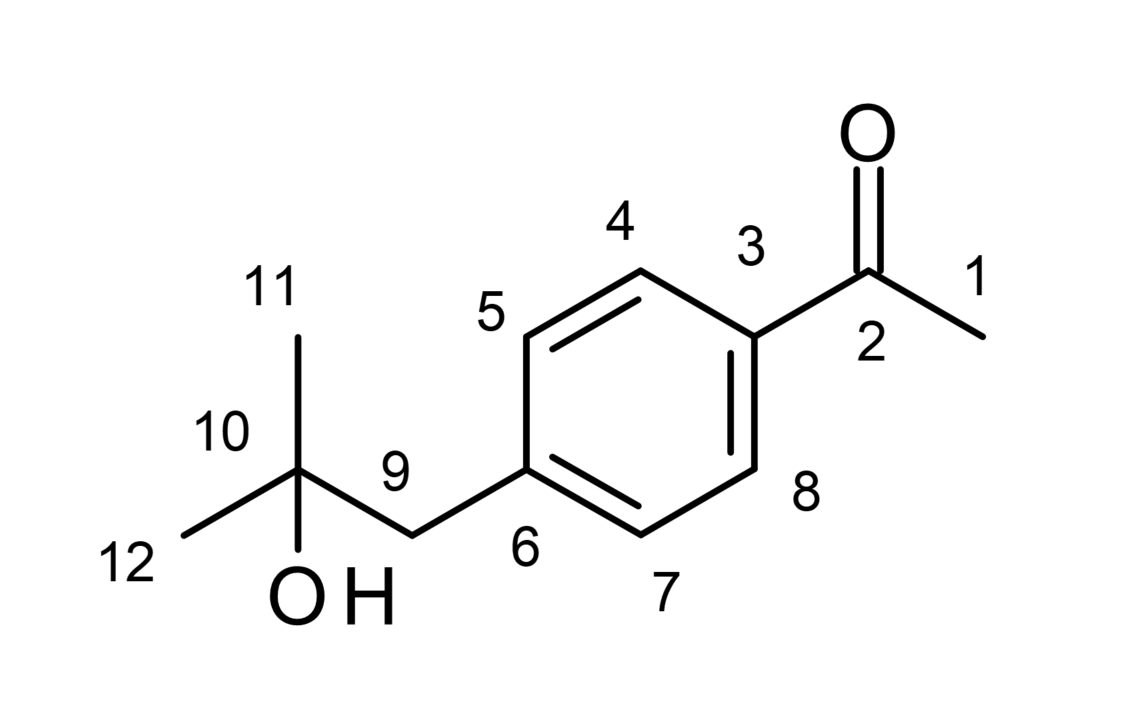 |
| 2.56 (s, 3H, H-1) | (128.0 (C-4, C-8)), (135.2 (C-3)), 198.0 (C-2) | 27.1 (C-1) |  |
| 2.74 (s, 2H, H-9) | 29.7 (C-11, C-12), 69.8 (C-10), 131.1 (C-5, C-7), 145.3 (C-6) | 49.7 (C-9) |  |
| 7.36 (d, J = 8.3 Hz, 2H, H-5, H-7) | 49.7 (C-9), 131.1 (C-5, C-7), 135.2 (C-3), (145.3 (C-6)), (198.0 (C-2)) | 131.1 (C-5, C-7) |  |
| 7.86 (d, J = 8.3 Hz, 2H, H-4, H-8) | 128.0 (C-4, C-8), 145.3 (C-6), 198.0 (C-2) | 128.0 (C-4, C-8) |  |
|  |  | 69.8 (C-10) |  |
|  |  | 135.2 (C-3) |  |
|  |  | 145.3 (C-6) |  |
|  |  | 198.0 (C-2) |  |

^1^ Chemical shifts are expressed in d (ppm) calibrated on the resonances of the residual nondeuterated solvent DMSO.

Table S6: NMR data and structure of 4-carboxy-α-methylbenzeneacetic acid^1^ (product P7) formed during the incubation of *Mycobacterium neoaurum* SBUG 109 with ibuprofen.

| ^1^H  Chemical shift^1^ (multiplicity, coupling constant, number of protons, proton assignment) | ^1^H-^13^C-correlation | ^13^C  Chemical shift^1^ (assignment of carbon atoms) | structure |
| --- | --- | --- | --- |
| 7.98 (d, J = 7.6 Hz, 2H, H-2/H-6) | 130.9 (C-2/C-6), 147.8 (C-4), 170.0 (C-7) | 130.9 (C-2/C-6) |  |
| 7.43 (d, J = 7.6 Hz, 2H, H-3/H-5) | 47.0 (C-8), 128.7 (C-3/C-5), 131.2 (C-1), (170.0 (C-7)) | 128.7 (C-3/C-5) |  |
| 3.80 (m, J = 7.2 Hz, 1H, H-8) | 18.9 (C-10), 128.7 (C-3/C-5), 147.8 (C-4), 178.0 (C-9) | 47.0 (C-8) |  |
| 1.49 (d, J = 7.2 Hz, 3H, H-10) | 47.0 (C-8), 147.8 (C-4), 178.0 (C-9) | 18.9 (C-10) |  |
|  |  | 131.2 (C-1) |  |
|  |  | 147.8 (C-4) |  |
|  |  | 170.0 (C-7) |  |
|  |  | 178.0 (C-9) |  |

^1^ Structure elucidation was carried out with the purified product formed during the transformation of an unidentified yeast strain isolated from sewage sludge

Table S8: Proteins with higher abundance in *Priestia megaterium* SBUG 518 cells harvested after 1 h and 24 h of incubation with 0.005% ibuprofen (IBU) compared to bacterial cells under control conditions without IBU. Bold numbers indicate statistically significant differences (FDR<0.05, log_2_ fold change ≥ 1). MMb: Mineral salts medium without IBU (control).

| **Accession number** | **Annotation** |  | **Fold change IBU:MMb 1h** | **Fold change IBU:MMb 24h** |
| --- | --- | --- | --- | --- |
| **Transporters** | |  |  |  |
| A0A0B6AF44 | HlyD secretion family protein | | **>15.00** | **>15.00** |
| A0A0B6ANT1 | HlyD secretion family protein | | **>15.00** | **>15.00** |
|  |  |  |  |  |
| **Cytochrome P450** | |  |  |  |
| P14779 | Bifunctional cytochrome P450/NADPH--P450 reductase (Cytochrome P450(BM-3)) | | 1.28 | **12.33** |
| A0A0B6ATP7 | Cytochrome P450 family protein | | 0.90 | **2.64** |
|  |  |  |  |  |
| **Lipid/propionate metabolism** | | |  |  |
| A0A0B6AEV5 | 3-hydroxypropionyl-coenzyme A dehydratase | | 7.46 | **>15.00** |
| A0A0B6AR85 | Enoyl-CoA hydratase/isomerase family protein | | 1.01 | **2.59** |
|  |  |  |  |  |
| **Amino acid metabolism** | |  |  |  |
| A0A0B6AEY8 | Histidinol dehydrogenase HisD | | 0.98 | **>15.00** |
| A0A0B6ANK4 | ARD/ARD' family protein | | 0.69 | **>15.00** |
| A0A0B6AZ96 | Phosphoserine aminotransferase SerC | | 0.89 | **2.32** |
| A0A0B6AFB6 | Imidazolonepropionase HutI | | 0.98 | **2.68** |
| A0A0B6ASH0 | Aminomethyltransferase GcvT | | 1.30 | **3.14** |
| A0A0B6AYG6 | Alanine dehydrogenase Ald | | 1.00 | **5.59** |
| A0A0B6AK49 | Ornithine aminotransferase RocD | | 1.37 | **2.71** |
|  |  |  |  |  |
| **Dehydrogenases** | | | |  |
| A0A0B6AMP6 | Iron-containing alcohol dehydrogenase family protein | | 0.89 | **>15.00** |
| A0A0B6AJ55 | Iron-containing alcohol dehydrogenase family protein | | 0.81 | **2.17** |
| A0A0B6ACL7 | Aldehyde dehydrogenase (NAD(+)) | | 0.98 | **2.33** |
| A0A0B6A5I2 | Acyl-CoA dehydrogenase | | 0.98 | **2.03** |
|  |  |  |  |  |
| **Carbohydrate metabolism** | | |  |  |
| A0A0B6A8K8 | 3-hexulose-6-phosphate isomerase HxlB | | 0.84 | **2.39** |
| A0A0B6ABH9 | Glutamine--fructose-6-phosphate aminotransferase [isomerizing] GlmS | | 0.87 | **2.06** |
| A0A0B6ASD9 | Citrate synthase CitA | | 1.13 | **2.12** |
|  |  |  |  |  |
| **Isoprenoid metabolism** | |  |  |  |
| A0A0B6AHS0 | 2-C-methyl-D-erythritol 2,4-cyclodiphosphate synthase IspF | | 0.72 | **2.96** |
|  |  |  |  |  |
| **Sporulation/septum formation** | | |  |  |
| A0A0B6AKZ7 | Probable septum site-determining protein MinC | | **>15.00** | **>15.00** |
| A0A0B6AGE1 | Putative septation protein SpoVG | | 0.91 | **2.75** |
|  |  |  |  |  |
| **Other** |  |  |  |  |
| A0A0B6AJG9 | Single-stranded-DNA-specific exonuclease RecJ | | 2.15 | **>15.00** |
| A0A0B6ARS0 | Zinc-binding dehydrogenase family protein | | 2.06 | **>15.00** |
| A0A0B6AWL4 | RNA polymerase sigma factor SigF | | 1.38 | **>15.00** |
| A0A0B6AZF4 | Flagellar motor switch protein FliN | | 0.83 | **>15.00** |
| A0A0B6AY49 | KduI/IolB family protein | | 0.95 | **4.38** |
| A0A0B6ACX4 | Thioesterase superfamily protein | | 0.93 | **2.04** |
| A0A0B6AEI7 | Oligopeptidase Pz-A | | 0.88 | **2.24** |
| A0A0B6AI89 | Sulfate adenylyltransferase Sat | | 1.02 | **2.49** |
| A0A0B6AQI7 | NAD kinase NadK | | 0.97 | **2.39** |
| A0A0B6AUL8 | DinB superfamily protein | | 0.93 | **3.30** |
| A0A0B6AHI3 | Relaxasome subunit MobC | | 1.68 | **10.12** |
|  |  |  |  |  |
| **Unknown function** | |  |  |  |
| A0A0B6AKK4 | DUF3243 domain-containing protein | | 0.92 | **>15.00** |
| A0A0B6AQM8 | Uncharacterized protein | | 0.61 | **2.00** |

Figure S1: Microscopic images (40:1, bar: 50 µm) of cultures of *Priestia megaterium* SBUG 518 after 120 h incubation. Incubation with ibuprofen in absence of glucose with (A) stationary-phase cells, and (B) logarithmic-phase cells as well as with ibuprofen in the presence of 0.1% glucose with (C) stationary-phase cells, and (D) logarithmic-phase cells.

# Supplementary Discussion

*Proteins potentially involved in IBU transformation by P. megaterium SBUG 518.* Iron-containing alcohol dehydrogenases (FeADHs) might generate ibuprofenal from 3-OH-IBU. Ibuprofenal could then be oxidized further to CBX-IBU via an aldehyde dehydrogenase. In *Clostridium*, FeADHs can have catalytic functions as propanediol dehydrogenase, butanol dehydrogenase or 4-hydroxybuyrate dehydrogenase (Youngleson et al., 1989; Wolff et al., 1993; Luers et al., 1997). Therefore, these enzymes utilize substrates similar to the isobutyl and propanoic acid residues of IBU, supporting our functional hypothesis.

Lipid/propionate metabolism might be involved in transformation of propionate to acetyl-CoA (via acrylate and 3-OH-propionate, as alternative to the methylmalonyl-CoA-pathway to succinyl-CoA), which could then be further metabolized via acetyl-CoA dehydrogenase. This is the case in several bacteria and eukaryotic organisms (Halarnkar et al., 1988; Wilson et al., 2017). Propionate can be split off from side chains of IBU by several bacteria (as shown e.g. in this study by the formation of 4-carboxy-α-methylbenzeneacetic acid by *M. neoaurum* SBUG 109). While in *P. megaterium* SBUG 518 we hitherto did not find C3-dealkylated products of IBU, at least four of the formed transformation products have not been identified yet. Alternatively, it cannot be ruled out that some of these enzymes could also directly transform the IBU side-chains without any off-splitting of C3-units or were induced during degradation of valine or isoleucine also resulting in propionyl-CoA formation. In the IBU-degrading, Gram-positive *Patulibacter* I11, proteins involved in fatty acid metabolism were also suggested to be involved in IBU degradation (Almeida et al., 2013).

*A pleiotropic effect of IBU on* P. megaterium *SBUG 518.* IBU seems to impact spore formation in *P. megaterium* SBUG 518. Corroborating this, we noticed that logarithmic-phase cells did not sporulate when incubated with IBU without glucose (data not shown). While data on the impact of IBU on cell wall turnover and sporulation seems to be lacking, several studies describe negative impacts of this drug on the membrane integrity of pro- and eukaryotic microbial species (Pina-Vaz et al., 2000; Lamsa et al., 2016; Ogundeji et al., 2016). The high resistance of *Bacillus thuringiensis* B1(2015b) against IBU, on the other hand, is likely based on changes in the membrane composition (Marchlewicz et al., 2017). In a broader context, sporulation in *B. subtilis* is inhibited by unsaturated fatty acids (Strauch et al., 1992), which bear structural and metabolic resemblance to IBU.

Additionally, DNA damage occurring in IBU presence was suggested by the presence of DNA exonuclease. In *Escherichia coli*, several NSAIDs impact DNA replication and repair by inhibition of DNA polymerase III beta subunit (Yin et al., 2014). It remains to be elucidated whether IBU has a similar mode of action in *P. megaterium* SBUG 518. Interestingly, based on relaxosome abundance, horizontal gene transfer might be promoted by IBU presence.

IBU also seems to interfere with the amino acid metabolism and protein synthesis of *P. megaterium* SBUG 518. Potential reasons for this are shifts in protein expression patterns, due to the synthesis of detoxifying and transporting proteins as well as repair proteins, and potential interference of IBU with gene expression. At the same time, in *P. megaterium* SBUG 518, the fatty acid metabolism is likely negatively impacted by IBU. While it remains unclear whether this is due to general energy shortage or due to direct effects of IBU in *P. megaterium* SBUG 518, also in oral pathogenic bacteria IBU potentially interacts with proteins involved in fatty acid metabolism (Vijayashree Priyadharsini, 2019).

The lower abundance of oxygen radical detoxifying enzymes in *P. megaterium* SBUG 518 in IBU presence was unexpected, as IBU has been shown to induce catalase activity in *P. megaterium* (English and Rankin, 1997), and cytochrome P450 activity leads to oxygen radical formation (Gagné et al., 2006).. However, reactive oxygen species themselves might be involved in cytochrome P450 BM3 induction, as induction in a *P. megaterium* strain decreased when cells were incubated with IBU and external catalase (English and Rankin, 1997). Therefore, protein expression regulation might be more complex than anticipated. Additionally, an antioxidant effect of IBU itself has been described (Costa et al., 2006), which might add to this protein abundance pattern. Moreover, IBU itself might interfere with the respective gene expression, actually exacerbating oxidative stress in *P. megaterium* SBUG 518.

Taken together, while *P. megaterium* SBUG 518 efficiently transforms IBU as a means of detoxification, the drug still elicits various and apparently detrimental effects on the physiology of this bacterium.

# Supplementary References

Almeida, B., Kjeldal, H., Lolas, I., Knudsen, A. D., Carvalho, G., Nielsen, K. L., et al. (2013). Quantitative proteomic analysis of ibuprofen-degrading *Patulibacter* sp. strain I11. *Biodegradation* 24, 615–630. doi: 10.1007/s10532-012-9610-5

Costa, D., Moutinho, L., Lima, J. L. F. C., and Fernandes, E. (2006). Antioxidant activity and inhibition of human neutrophil oxidative burst mediated by arylpropionic acid non-steroidal anti-inflammatory drugs. *Biol. Pharm. Bull.* 29, 1659–1670.

De Boer, T. D., and Backer, H. J. (1956). “Diazomethane,” in *Organic synthesis* , ed. L. N. J. (Wiley, New York, NY), 14–16.

English, N. T., and Rankin, L. C. (1997). Antioxidant-mediated attenuation of the induction of cytochrome P450BM-3(CYP102) by ibuprofen in *Bacillus megaterium* ATCC 14581. *Biochem. Pharmacol.* 54, 443–450. doi: 10.1016/S0006-2952(97)00054-3

Gagné, F., Blaise, C., and André, C. (2006). Occurrence of pharmaceutical products in a municipal effluent and toxicity to rainbow trout (*Oncorhynchus mykiss*) hepatocytes. *Ecotoxicol. Environ. Saf.* 64, 329–336. doi: 10.1016/j.ecoenv.2005.04.004

Halarnkar, P. P., Wakayama, E. J., and Blomquist, G. J. (1988). Metabolism of propionate to 3-hydroxypropionate and acetate in the lima bean *Phaseolus limensis*. *Phytochemistry* 27, 997–999. doi: 10.1016/0031-9422(88)80259-0

Lamsa, A., Lopez-Garrido, J., Quach, D., Riley, E. P., Pogliano, J., and Pogliano, K. (2016). Rapid inhibition profiling in *Bacillus subtilis* to identify the mechanism of action of new antimicrobials. *ACS Chem. Biol.* 11, 2222–2231. doi: 10.1021/acschembio.5b01050

Luers, F., Seyfried, M., Daniel, R., and Gottschalk, G. (1997). Glycerol conversion to 1,3-propanediol by *Clostridium pasteurianum*: Cloning and expression of the gene encoding 1,3-propanediol dehydrogenase. *FEMS Microbiol. Lett.* 154, 337–345. doi: 10.1016/S0378-1097(97)00351-0

Marchlewicz, A., Guzik, U., Hupert-Kocurek, K., Nowak, A., Wilczyńska, S., and Wojcieszyńska, D. (2017). Toxicity and biodegradation of ibuprofen by *Bacillus thuringiensis* B1(2015b). *Environ. Sci. Pollut. Res.* 24, 7572–7584. doi: 10.1007/s11356-017-8372-3

Ogundeji, A. O., Pohl, C. H., and Sebolai, O. M. (2016). Repurposing of aspirin and ibuprofen as candidate anti-*Cryptococcus* drugs. *Antimicrob. Agents Chemother.* 60, 4799–4808. doi: 10.1128/AAC.02810-15

Pina-Vaz, C., Sansonetty, F., Rodrigues, A. G., Martinez-de-Oliveira, J., Fonseca, A. F., and Mårdh, P.-A. (2000). Antifungal activity of ibuprofen alone and in combination with fluconazole against *Candida* species. *J. Med. Microbiol.* 49, 831–840. Available at: http://www.ncbi.nlm.nih.gov/pubmed/10966233

Strauch, M. A., de Mendoza, D., and Hoch, J. A. (1992). *cis*‐Unsaturated fatty acids specifically inhibit a signal‐transducing protein kinase required for initiation of sporulation in *Bacillus subtilis*. *Mol. Microbiol.* 6, 2909–2917. doi: 10.1111/j.1365-2958.1992.tb01750.x

Vijayashree Priyadharsini, J. (2019). In silico validation of the non-antibiotic drugs acetaminophen and ibuprofen as antibacterial agents against red complex pathogens. *J. Periodontol.* 90, 1441–1448. doi: 10.1002/JPER.18-0673

Wilson, K. A., Han, Y., Zhang, M., Hess, J. P., Chapman, K. A., Cline, G. W., et al. (2017). Inter-relations between 3-hydroxypropionate and propionate metabolism in rat liver: Relevance to disorders of propionyl-CoA metabolism. *Am. J. Physiol. - Endocrinol. Metab.* 313, E413–E428. doi: 10.1152/ajpendo.00105.2017

Wolff, R. A., Urben, G. W., O’Herrin, S. M., and Kenealy, W. R. (1993). Dehydrogenases involved in the conversion of succinate to 4-hydroxybutanoate by *Clostridium kluyveri*. *Appl. Environ. Microbiol.* 59, 1876–1882.

Yin, Z., Wang, Y., Whittell, L. R., Jergic, S., Liu, M., Harry, E., et al. (2014). DNA replication is the target for the antibacterial effects of nonsteroidal anti-inflammatory drugs. *Chem. Biol.* 21, 481–487. doi: 10.1016/j.chembiol.2014.02.009

Youngleson, J. S., Jones, W. A., Jones, D. T., and Woods, D. R. (1989). Molecular analysis and nucleotide sequence of the adh1 gene encoding an NADPH-dependent butanol dehydrogenase in the Gram-positive anaerobe *Clostridium acetobutylicum*. *Gene* 78, 355–364. doi: 10.1016/0378-1119(89)90238-2

Zühlke, M.-K., Schlüter, R., Mikolasch, A., Zühlke, D., Giersberg, M., Schindler, H., et al. (2017). Biotransformation and reduction of estrogenicity of bisphenol A by the biphenyl-degrading *Cupriavidus basilensis*. *Appl. Microbiol. Biotechnol.* 101, 3743–3758. doi: 10.1007/s00253-016-8061-z
